# Nomogram for predicting survival in patients with mucinous breast cancer undergoing chemotherapy and surgery: a population-based study

**DOI:** 10.1186/s40001-023-01395-x

**Published:** 2023-10-10

**Authors:** Ting Gao, Yuyuan Chen, Ming Li, Keying Zhu, Rong Guo, Yiyin Tang, Sheng Huang, Dedian Chen

**Affiliations:** 1 The 2Nd Department of Breast Surgery, Breast Cancer Center of the Third Affiliated Hospital of Kunming Medical University, Yunnan Cancer Hospital, Building 3, No. 519 Kunzhou Road, Kunming, 650118 China; 2 The Department of Thyroid and Breast Surgery, Dali Bai Autonomous Prefecture People‘s Hospital, Dali, 671000 China; 3 The Department of Thyroid and Breast Surgery, The Affiliated Hospital of Ningbo University Medical College, Ningbo, 315000 China; 4 The Department of General Surgery, Qujing Maternal and Child Health-Care Hospital, Qujing, 655000 China

**Keywords:** Mucinous breast cancer, Prognostic factors, Predict, Nomogram, Survival

## Abstract

**Background:**

The prognosis of patients with mucinous breast cancer (MuBC) is affected by several factors, but the low incidence of MuBC makes it difficult to conduct extensive and in-depth studies. This study was designed to establish a prognostic model and verify its accuracy in patients with MuBC after chemotherapy and surgery to help develop personalized treatment strategies.

**Materials and methods:**

Patients with MuBC who underwent chemotherapy and surgery from 2004 to 2015 were identified in the Surveillance, Epidemiology, and End Results (SEER) database. The prognostic factors of patients with MuBC were investigated using a Cox proportional hazards regression analysis. Based on the identified factors, a nomogram was constructed to forecast the overall survival (OS) of patients at 3, 5, and 10 years. Internal (from SEER) and external (from Yunnan Cancer Center, YNCC) verification queues were used to verify the nomogram and demonstrate the predictive capacity of this model.

**Results:**

The study comprised 1668 MuBC patients from the SEER database and 107 from the YNCC. The nomogram included four characteristics: age, anatomical stage, surgical method, and radiotherapy. The concordance indices in the training, internal verification, and external verification queues were 0.680, 0.768, and 0.864, respectively. The calibration curves for the nomogram showed excellent agreement between the predictions and observations. This nomogram has good clinical application value according to the decision curve analysis.

**Conclusions:**

The prognosis of patients with MuBC who have undergone chemotherapy and surgery can be forecasted using this nomogram, which would be beneficial to help create individualized treatment plans for the affected patients.

## Introduction

Breast cancer (BC) has high incidence and mortality rates. In 2020, the incidence of BC was the highest worldwide, and was also the primary cause of cancer-related deaths in women [1]. Mucinous breast cancer (MuBC) is a special type of invasive BC that is relatively rare, accounting for approximately 2.6% of cases of invasive BC [2]. MuBC is common in perimenopausal or postmenopausal women and has a favorable prognosis. The main characteristic of MuBC is an abundance of extracellular mucin, and tumor cells float in the extracellular mucus as nests or islands [3]. There are two subtypes of MuBC: pure MuBC, containing at least 90% mucin, and mixed MuBC, containing less than 90% mucin and other types of BC [4]. Pure MuBC offers a favorable prognosis as well as an 80–100% 10-year survival rate, while the prognosis of mixed MuBC depends on the biological characteristics of the other carcinomas in the mixed component [3].

MuBC has a higher positivity rate for hormone receptors (HR), less lymph node metastasis, lower anatomical stage, and less human epidermal growth factor receptor 2 (HER-2) overexpression than invasive ductal carcinoma (IDC) [5]. There are many prognostic factors for MuBC; lymph node involvement (N) is the most significant factor, followed by the age at diagnosis, tumor size (T), progesterone receptor (PR) status, and nuclear grade [6]. The rarity of MuBC has hindered the extensive evaluation of its treatment and prognostic factors. Current treatments are mostly derived from small-sample studies or refer to the IDC's treatment experience. To the best of our knowledge, there is no standard prognostic assessment system for MuBC, although numerous studies have confirmed its prognostic factors.

A nomogram can integrate various prognostic factors into a statistical model that then generates the numerical probability of a clinical event and is widely used to estimate cancer prognosis and survival [7]. Presently, several clinical nomograms have been developed for various types of BC [8–11]. However, a nomogram for predicting the survival of patients with MuBC after chemotherapy and surgery has not been constructed. Therefore, we created and validated a nomogram that predicts the survival of this population using information from the Surveillance, Epidemiology, and End Results (SEER) database. We also retrospectively collected data from the Yunnan Cancer Center (YNCC) database to identify the effectiveness of this new nomogram. The aim of this study was to establish a prognostic model and verify its accuracy in patients with MuBC after chemotherapy and surgery to provide personalized medical services for this population.

## Materials and methods

### Patient selection

In this study, patient information was gathered from two sources. First, we searched the SEER database for MuBC (histological subtype code 8480/3, ICD-O-3) patient information from 2004 to 2015. The following exclusion criteria were used: (1) male; (2) patients with other malignancies in addition to BC; (3) patients with bilateral breast cancer; (4) patients treated without chemotherapy and surgery; (5) patients who lacked vital clinical information (including HR status, TNM stage, and radiotherapy information); and (6) patients with missing or unknown survival time. Finally, 1668 qualified patients were enrolled in our research and separated into a training queue (n = 1169) and an internal verification queue (n = 499) in a 7:3 ratio. Furthermore, we retrospectively collected data from 107 eligible patients with MuBC at the YNCC between January 2008 and September 2021 for external verification.

### Data collection

Demographic information of patients collected from the SEER and YNCC databases included age at diagnosis, estrogen receptor (ER) status, PR status, HER-2 status, anatomical stage, historic stage, radiotherapy, and surgical method. Patient survival information was obtained by reviewing medical records and telephone follow-ups, and the overall survival (OS) was calculated. OS is defined as the period from histological diagnosis to death (regardless of the cause of death) or the final follow-up. Patients were divided into anatomical stages I, II, III, and IV according to the American Joint Committee on Cancer (AJCC) TNM staging criteria (8th edition). MuBC subtypes were classified according to ER, PR, and HER-2 status, and included HR+/HER2+, HR+/HER2−, HR-/HER2+, and HR-/HER2−. X-tile software may transform continuous variables into categorical variables by determining the most appropriate cut-off values [12]. A cut-off age of 52 years was determined using the X-tile software. Patients were divided into two groups according to age: young (≤ 52 years) and old (> 52 years).

### Statistical analysis

Percentages and frequencies were used to express the categorical variables. A survival curve was generated using the Kaplan–Meier method, and a log-rank test was used to assess differences in survival among the groups. The variables associated with OS were identified using univariate Cox proportional hazards regression analysis. Significant variables (*P* < 0.05) determined by univariate analysis were incorporated into the multivariable Cox proportional hazards regression analysis to determine independent prognostic factors for MuBC [13]. Based on the identified factors, a nomogram was created to forecast the OS of patients over 3, 5, and 10 years using the "rms" package in the R language. A receiver operating characteristic (ROC) curve was constructed to evaluate the accuracy of the nomogram using the area under the curve (AUC). The concordance index (C-index) was obtained using the four packages "survival, ggpubr, survminer, ggplot" in R language, which was used to assess the performance of the prediction model. The model is more accurate the closer its C-index and AUC are to 1. When the value is higher than 0.7, the model is generally regarded as being reliable [14]. The consistency between the predicted and observed values was evaluated using the calibration curves. And calibration curves were obtained using the "rms, foreign and survival" packages in R and used to assess the accuracy of the model predictions. Decision curve analysis (DCA) was performed using the R language "rms, foreign and survival" packages to clarify the clinical utility of the predictive model. Each independent prognostic factor was scored, and the scores for each factor were added to obtain a patient risk score based on the constructed nomogram. Using the median risk score for all patients in the queue as a boundary, patients were divided into high- and low-risk groups, and a log-rank test was applied to compare groups. We verified the nomogram performance both internally and externally to fully assess the forecasting ability of the model.

The test level of this study was α = 0.05, and all *P*-values were two-tailed. SPSS (version 25.0) and R (version 4.1.1) were used for all statistical analyses. And the symbol "*" indicates: *P* < 0.05.

## Results

### Clinicopathological characteristics of the population

A total of 1668 MuBC patients from the SEER database and 107 MuBC patients from the YNCC were included in this study. Of these, 1169 (70%) patients from the SEER database were randomly selected for the training queue. Clinicopathological features of the patients are presented in Table 1. An age cut-off was defined at 52 years, and the study population was divided into young and old groups. The training queue was split almost evenly between the young and old groups, at 49.30% and 50.70%, respectively. The most common molecular subtype in the training queue was HR+/HER2− (33.40%), vastly outnumbering the other three subtypes. Patients positive for ER (93.80%) and PR (80.80%) were in the majority; the HER-2 status was known in 46.40% of patients, including 12.20% positive and 34.20% negative. Early-stage MuBC was detected in 83.20% of patients (approximately 32.70% in stage I and 50.50% in stage II), and 63.40% of patients did not have lymph node metastasis. In terms of surgical methods, 48.00% of patients opted for breast-conserving surgery and 57.30% received radiotherapy. The YNCC queue had more people in the young group, accounting for 72.00%; 52.30% of patients presented with HR+/HER2−, and 74.80% of patients chose modified radical surgery.

**Table 1:** Characteristics of patients with MuBC after chemotherapy and surgery

Variables	Total queue(SEER)		Training queue		Internal verification queue		External verification queue	
	*N* = 1668		*N* = 1169		*N* = 499		*N* = 107	
	*n*	%	*n*	%	*n*	%	*n*	%
Age								
≤ 52	835	50.1	576	49.3	259	51.9	77	72.0
> 52	833	49.9	593	50.7	240	48.1	30	28.0
Breast subtype								
HR+/HER-2−	544	32.6	390	33.4	154	30.9	56	52.3
HR + /HER2+	178	10.7	126	10.8	52	10.4	10	9.3
HR-/HER-2+	25	1.5	17	1.5	8	1.6	2	1.9
HR-/HER-2−	14	0.8	10	0.9	4	0.8	2	1.9
Unknown	907	54.4	626	53.6	281	56.3	37	34.6
ER status								
Negative	104	6.2	72	6.2	32	6.4	9	8.4
Positive	1564	93.8	1097	93.8	467	93.6	98	91.6
PR status								
Negative	332	19.9	225	19.2	107	21.4	16	15.0
Positive	1336	80.1	944	80.8	392	78.6	91	85.0
HER2 status								
Negative	558	33.5	400	34.2	158	31.7	58	54.2
Positive	203	12.2	143	12.2	60	12.0	12	11.2
Unknown	907	54.4	626	53.6	281	56.3	37	34.6
Stage								
I	540	32.4	382	32.7	158	31.7	30	28.0
II	836	50.1	590	50.5	246	49.3	53	49.5
III	268	16.1	179	15.3	89	17.8	22	20.6
IV	24	1.4	18	1.5	6	1.2	2	1.9
T stage								
1	700	42.0	489	41.8	211	42.3	40	37.4
2	669	40.1	465	39.8	204	40.9	59	55.1
3	211	12.6	153	13.1	58	11.6	6	5.6
4	64	3.8	44	3.8	20	4.0	2	1.9
Unknown	24	1.4	18	1.5	6	1.2	0	0
N stage								
0	1049	62.9	741	63.4	308	61.7	66	61.7
1	458	27.5	325	27.8	133	26.7	23	21.5
2	101	6.1	59	5.0	42	8.4	8	7.5
3	60	3.6	44	3.8	16	3.2	10	9.3
M stage								
0	1644	98.6	1151	98.5	493	98.8	105	98.1
1	24	1.4	18	1.5	6	1.2	2	1.9
Historic stage								
Localized	1023	61.3	726	62.1	297	59.5	/	/
Regional	593	35.6	408	34.9	185	37.1	/	/
Distant	52	3.1	35	3.0	17	3.4	/	/
Surgery								
BCS	792	47.5	561	48.0	231	46.3	24	22.4
Mastectomy	418	25.1	292	25.0	126	25.3	3	2.8
Radical	458	27.5	316	27.0	142	28.5	80	74.8
Radiotherapy								
No	701	42.0	499	42.7	202	40.5	53	49.5
Yes	967	58.0	670	57.3	297	59.5	54	50.5
Survival								
Alive	1423	85.3	1001	85.6	422	84.6	100	93.5
Dead	245	14.7	168	14.4	77	15.4	7	6.5

*HR* hormone receptors, *HER-2* human epidermal growth factor receptor 2, *ER* estrogen receptor, *PR* progesterone receptor, *BCS* breast-conserving surgery

According to the survival information obtained from the SEER database, the median survival time was 99.00 (1–179) months in the total queue, 97.00 (1–179) months in the training queue and 101.00 (9–179) months in the internal validation queue. The survival rates were 85.30% (1423/1668), 85.60% (1001/1169) and 84.60% (422/499) in the total queue, training queue and internal validation queue, respectively. By follow-up, the median survival time of the YNCC queue was 87.00 (13–191) months and survival rate was 93.50% (100/107).

### Prognostic factors in MuBC patients

**Fig. 1:**
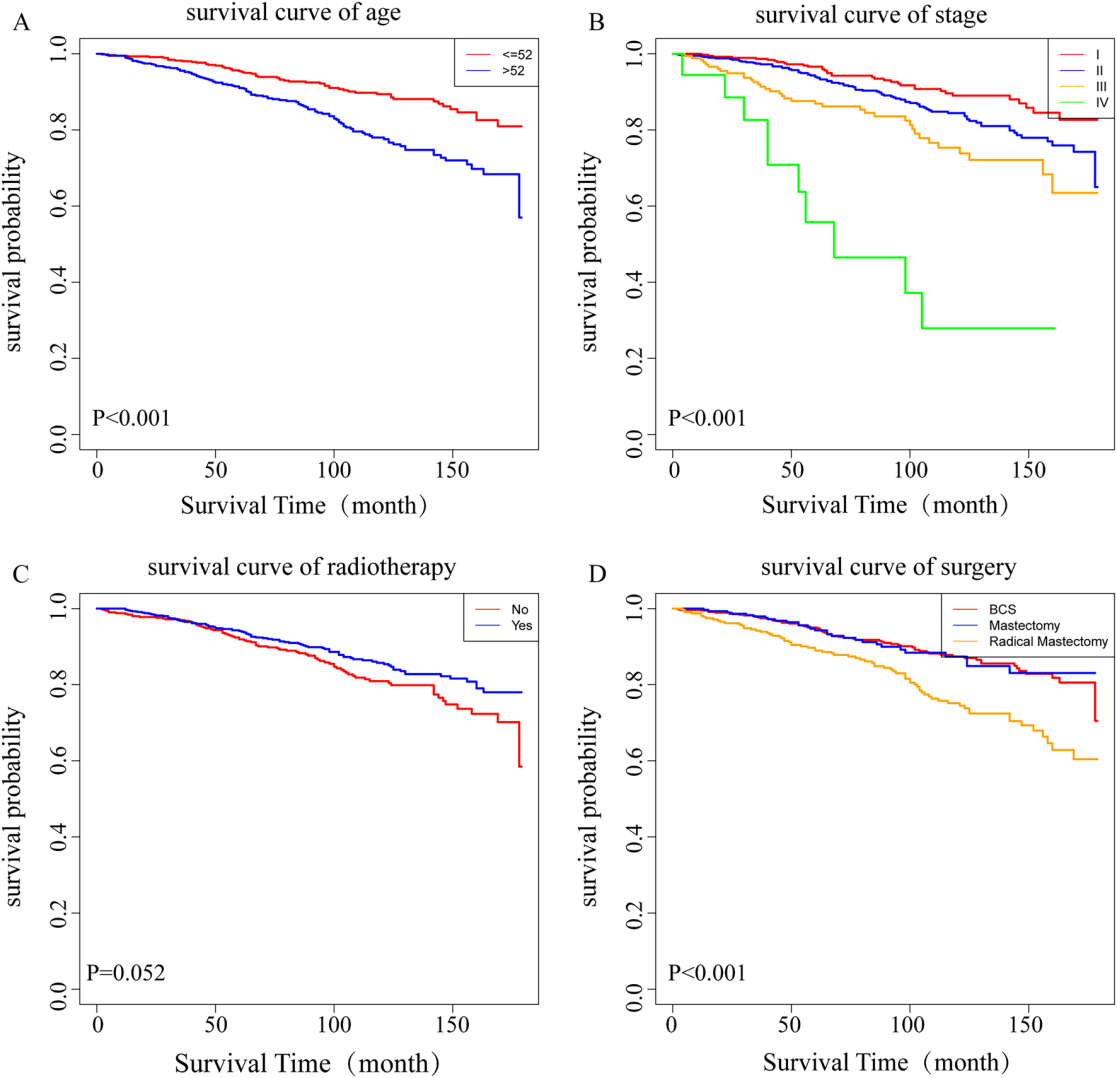
Kaplan–Meier curves of the OS stratified by age (**A**), stage (**B**), radiotherapy (**C**), surgical method (**D**)

A Cox proportional hazards regression model was used to investigate the prognostic factors of patients with MuBC in the training queue. Univariate analysis showed that age, anatomical stage, T stage, N stage, M stage, surgical method, radiotherapy, and historic stage were prognostic factors for OS (*P* < 0.05). These factors were incorporated into a multivariable analysis to investigate independent prognostic factors for MuBC. The results indicated that age and anatomical stage were independent factors affecting MuBC prognosis. Age > 52 years (HR: 1.980; 95%CI:1.437–2.728, *P* < 0.001) was associated with poor prognosis and was considered a risk factor. Compared with stage I patients, those with stages II (HR: 1.682, 95%CI: 1.097–2.580, *P* = 0.017), III (HR: 2.752, 95%CI: 1.382–5.479, *P* = 0.004) and IV (HR: 6.642, 95%CI: 1.962–22.491, *P* = 0.002) had worse prognoses. Surgical method and radiotherapy were important prognostic factors for MuBC (Table 2). Kaplan–Meier plots of the OS of patients with MuBC were drawn according to the results of the multivariable analysis, and the variations among groups were analyzed using the log-rank test (Fig. 1). The results showed that age > 52 years (*P* < 0.001), advanced stage (*P* < 0.001), and lack of radiotherapy (*P* = 0.052) were significantly associated with a poor prognosis. Breast-conserving surgery and mastectomy did not result in different survival rates, but both were superior to patients who underwent modified radical mastectomy (*P* < 0.001).

**Table 2:** Cox analysis of prognostic factors in MuBC patients in the training queue

Characteristics	Univariate analysis		Multivariable analysis	
	HR (95% CI)	*P*-value	HR (95% CI)	*P*-value
Age				
≤ 52	Reference		Reference	
> 52	2.081 (1.514–2.862)	< 0.001^*^	1.980 (1.437–2.728)	< 0.001^*^
Breast subtype				
HR+/HER-2−	Reference			
HR+/HER-2+	1.028 (0.505–2.091)	0.940		
HR−/HER-2+	0.613 (0.084–4.490)	0.630		
HR−/HER-2−	0.000 (0.000–infinity)	0.951		
Unknown	1.016 (0.670–1.541)	0.940		
Stage				
I	Reference		Reference	
II	1.625 (1.097–2.408)	0.015^*^	1.682 (1.097–2.580)	0.017^*^
III	2.684 (1.690–4.262)	< 0.001^*^	2.752 (1.382–5.479)	0.004^*^
IV	10.772 (5.321–21.808)	< 0.001^*^	6.642 (1.962–22.491)	0.002^*^
ER status				
Negative	Reference			
Positive	0.634 (0.373–1.077)	0.092		
PR status				
Negative	Reference			
Positive	0.830 (0.576–1.195)	0.316		
HER-2 status				
Negative	Reference			
Positive	0.994 (0.501–1.972)	0.986		
Unknown	1.044 (0.688–1.583)	0.841		
Historic stage				
Localized	Reference		Reference	
Regional	1.309 (0.946–1.811)	0.104	0.754 (0.483–1.177)	0.213
Distant	5.382 (3.158–9.172)	< 0.001^*^	1.449 (0.535–3.925)	0.466
Surgery				
BCS	Reference		Reference	
Mastectomy	0.997 (0.643–1.545)	0.989	0.779 (0.478–1.270)	0.316
Radical	2.069 (1.483–2.887)	< 0.001^*^	1.343 (0.893–2.019)	0.157
Radiotherapy				
No	Reference		Reference	
Yes	0.741 (0.548–1.003)	0.053	0.727 (0.511–1.036)	0.078

*HR* hormone receptors, *HER-2* human epidermal growth factor receptor 2, *ER* estrogen receptor, *PR* progesterone receptor, *BCS* breast-conserving surgery

*P < 0.05

### Prognostic nomogram construction and verification

**Fig. 2:**
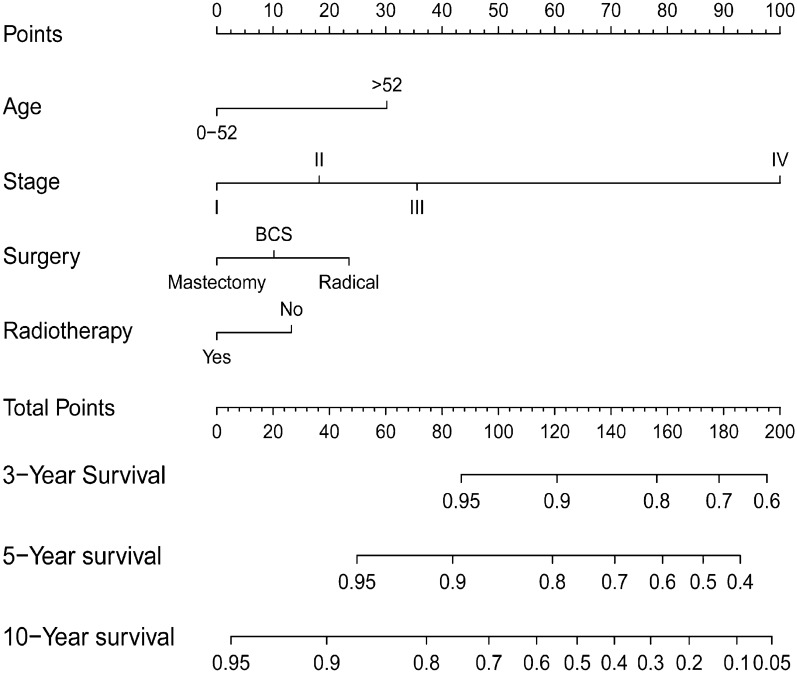
The nomogram that was employed to forecast the probability of the OS for patients with MuBC. Instructions for use: each variable corresponds to a fraction on the top point axis. Add the scores for each variable and find the point corresponding to the total score on the total point axis at the bottom. The total score was projected onto the survival axis to obtain the probability of the OS of a patient at 3, 5, and 10 years.

**Fig. 3:**
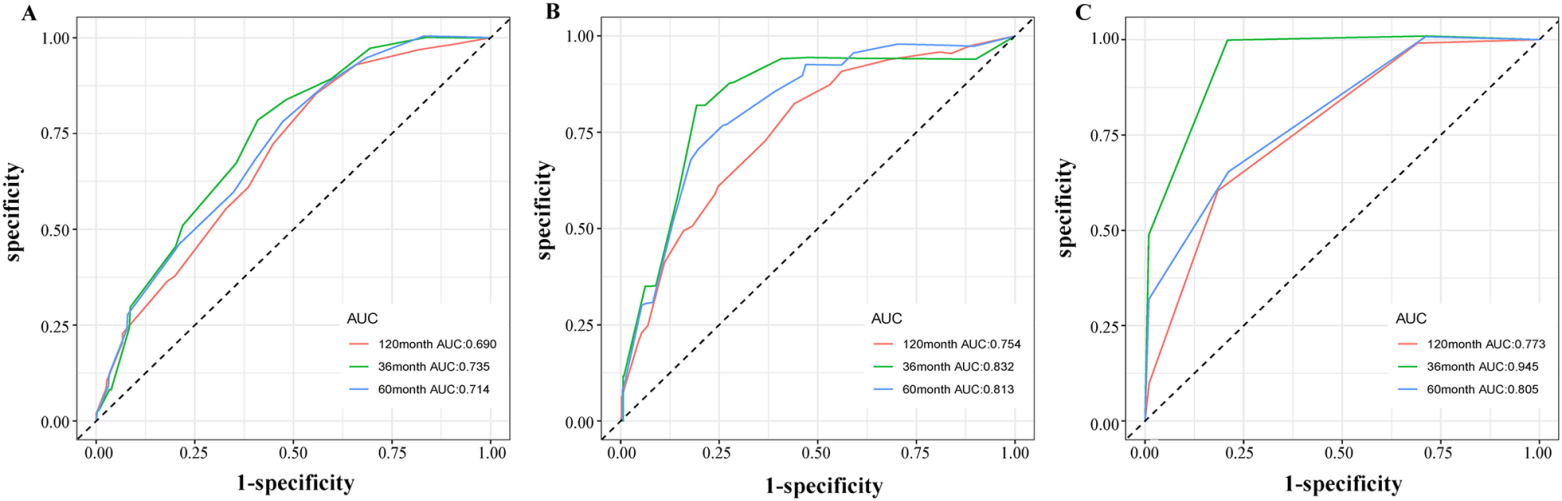
ROC curves of the nomogram were used to predict the OS at 3, 5, and 10 years in the training queue (**A**), internal verification queue (**B**), and external verification queue (**C**)

**Fig. 4:**
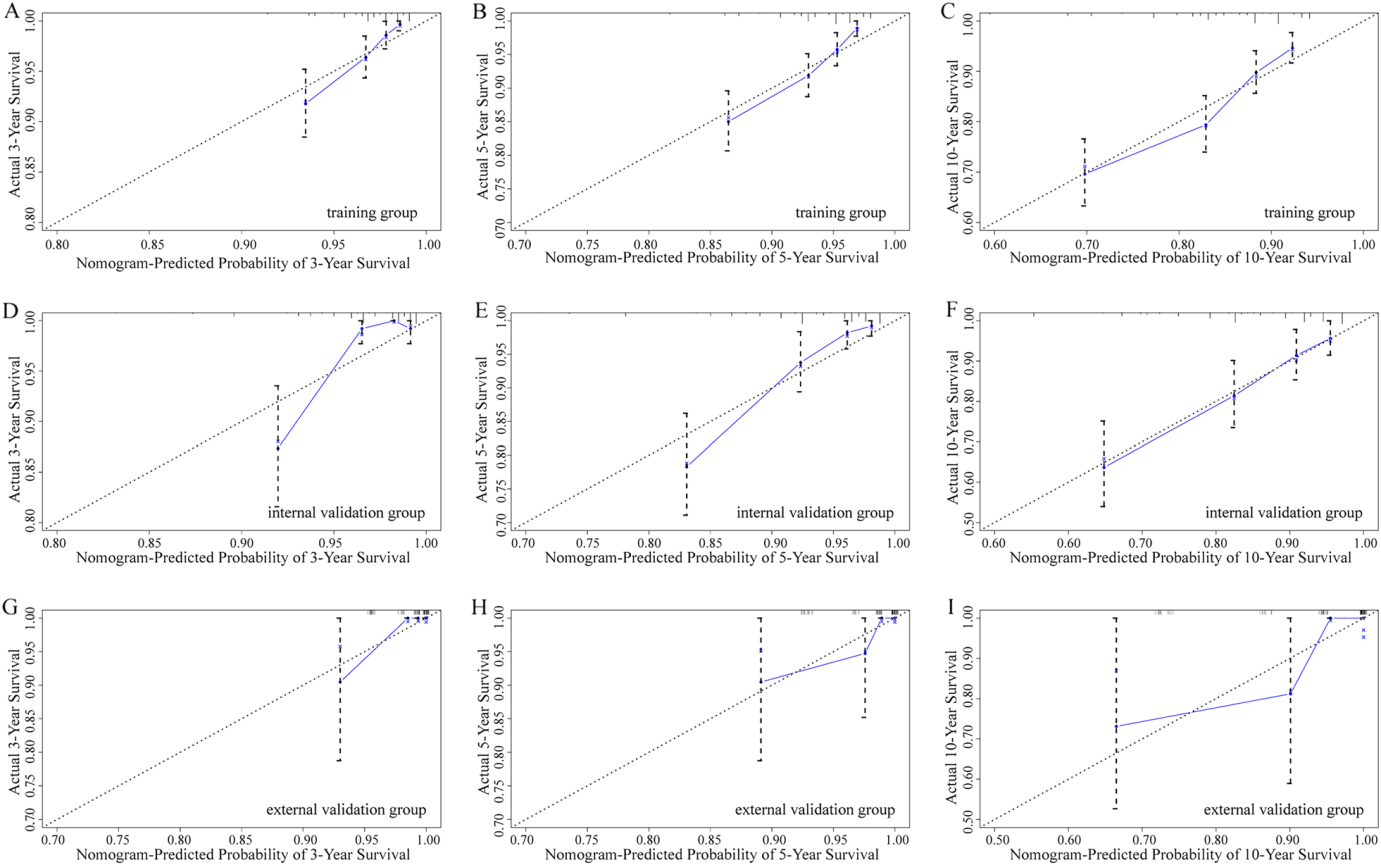
The calibration curves of the nomogram for the 3-, 5-, and 10-year OS prediction of the training queue (**A**–**C**), internal verification queue (**D**–**F**), and external verification queue (**G**–**I**). The OS probability predicted by the nomogram is plotted on the *X*-axis, and the actual probability is plotted on the *Y*-axis. The dotted line (plotted by *y* = *x*) shows the agreement of the actual result with the predicted result

**Fig. 5:**
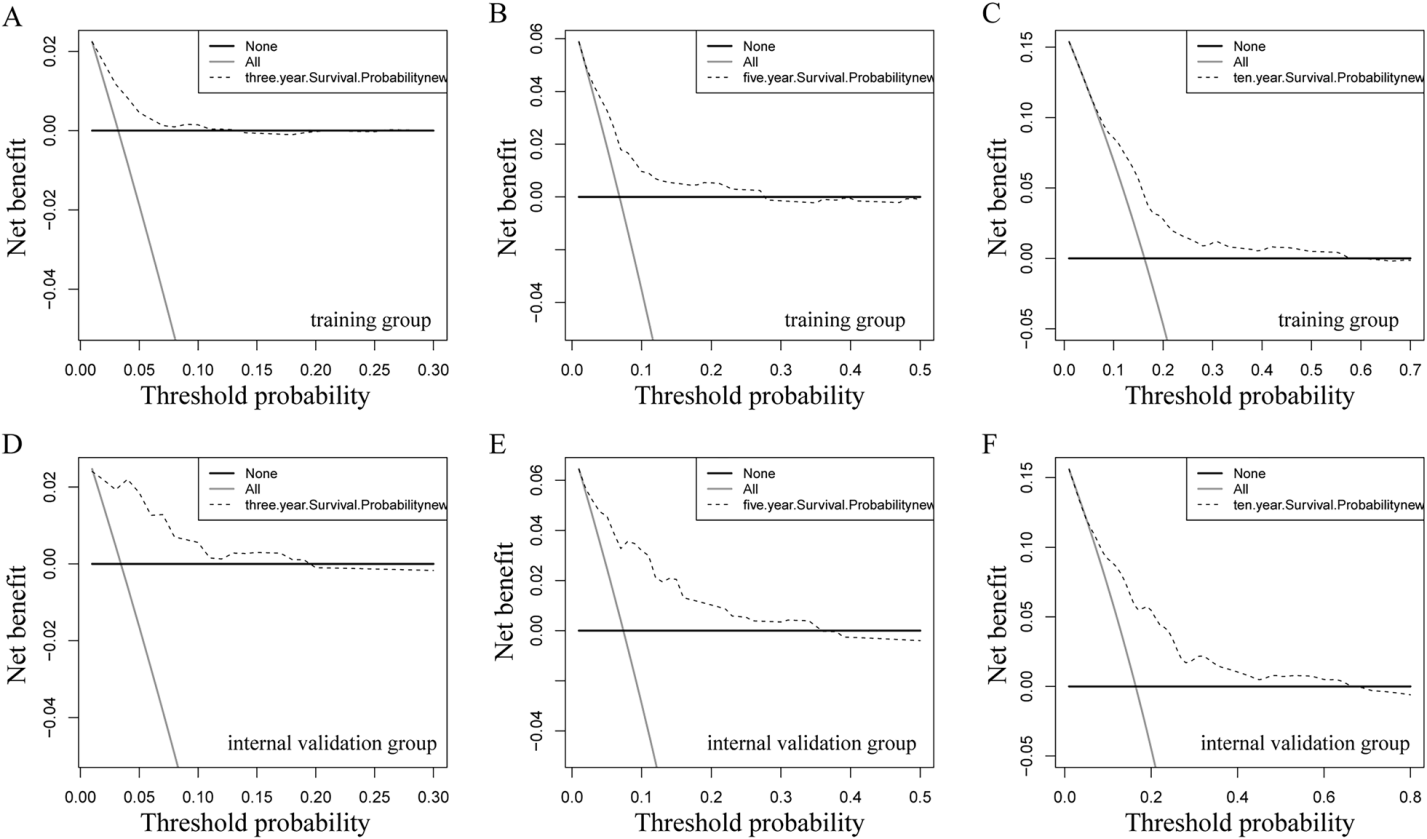
The DCA of the nomogram for the 3-, 5-, and 10-year OS prediction of the training queue (**A**–**C**) and internal verification queue (**D**–**F**)

Using the determined prognostic variables, a survival nomogram of patients with MuBC was constructed for OS at 3, 5, and 10 years (Fig. 2). The nomogram showed that the most significant contributor to the prognosis was stage, followed by age, surgical method, and radiotherapy. An ROC curve was used to assess the accuracy of the nomogram. The AUC values for the OS at 3, 5, and 10 years were 0.735, 0.714, and 0.690 in the training queue and 0.832, 0.813, and 0.754 in the internal verification queue, respectively (Fig. 3). The C-index of the nomogram for OS was 0.680 (95% CI:0.641–0.719) in the training queue and 0.768 (95% CI: 0.713–0.823) in the internal verification queue. The calibration curves for the nomogram showed excellent agreement between the actual observations and predictions (Fig. 4). In the training queue, all calibration curves had a high degree of coincidence with the reference line. In the internal verification queue, the 10-year calibration curve was in good agreement with the reference line, whereas the 3-year coincidence degree was relatively poor. The results of DCA showed that this prognostic model can be used to obtain a good net profit within a certain threshold range (Fig. 5).

Furthermore, we used data from the YNCC as an external verification queue to further validate the constructed nomogram. The AUC values for the OS at 3, 5, and 10 years were 0.945, 0.805, and 0.773, respectively. The C-index for the nomogram was 0.864 (95% CI: 0.754–0.974). The calibration curves showed some difference between the predictions and observations, which was likely caused by the short sample size of the YNCC cohort. The DCA graph could not be obtained due to data reasons (both the sample size and the number of outcome events were too small).

**Fig. 6:**
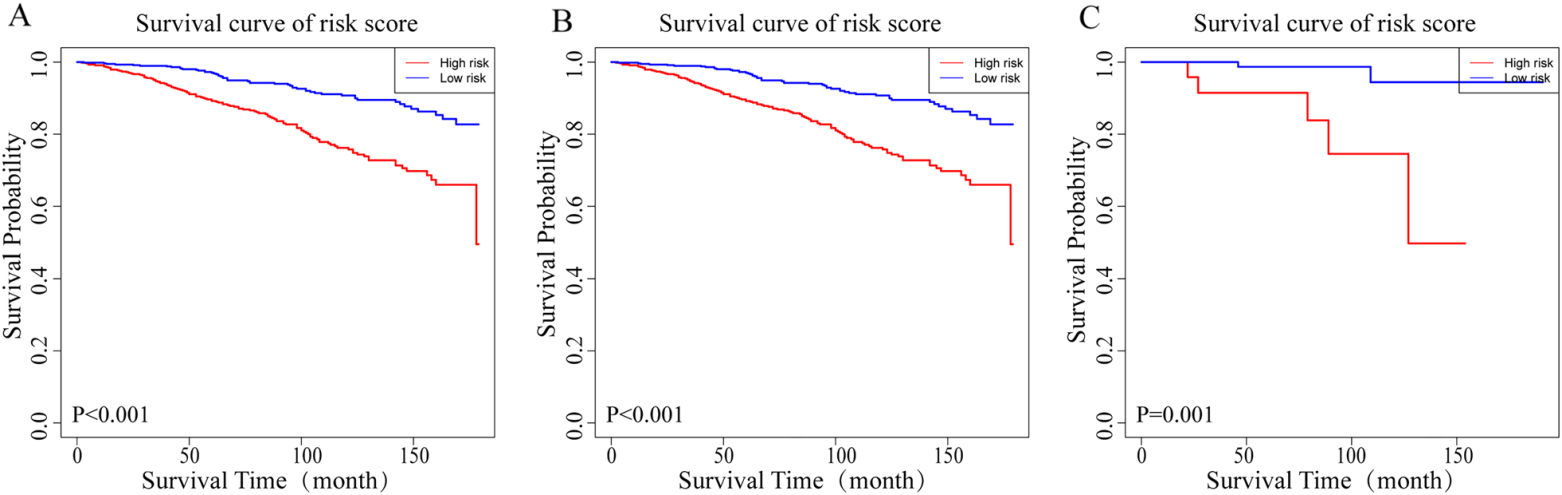
Kaplan–Meier survival analysis curves for the training queue (**A**), internal verification queue (**B**), and external verification queue (**C**), stratified by risk

The patients were divided into high- and low-risk groups based on the risk score obtained by the nomogram, and Kaplan–Meier survival analysis showed that the high-risk group had a worse prognosis. In the training queue (*P* < 0.001), internal verification queue (*P* < 0.001), and external verification queue (*P* = 0.001), significant disparities in the OS between the high- and low-risk groups were observed, with the OS of the high-risk group being significantly shorter (Fig. 6).

## Discussion

Several prognostic variables for MuBC were identified in this study, and the survival nomogram of MuBC patients after chemotherapy and surgery were constructed and validated, which accurately predicted the OS of patients at 3, 5, and 10 years. Molecular phenotype is an independent prognostic factor for MuBC (*P* < 0.05) [15]. Our study suggests that HR status is not an independent prognostic factor for OS in patients with MuBC. We believe this was related to the high positivity rate of HR in the study population, thus masking the impact of HR status on survival. Gwark et al. noted that HER-2 overexpression is an independent risk factor for MuBC [16]. Our study did not show that HER-2 status was related to the prognosis of MuBC, since the HER-2 status was unclear in more than half of the patients, and overexpression was detected in only approximately 12% of patients.

The AJCC classifies BCstages based on T, N, and distant metastasis (M) to help clinicians clarify the extent of the disease, determine treatment plans, and assess prognosis [17]. Komenaka et al. recommended that tumor size should not be an independent prognostic factor for MuBC because a large amount of mucin is included in the evaluation of tumor size [18]. However, a larger tumor load was thought to be associated with lymph node involvement (*P* = 0.0003) [19]. Lymph node metastasis is an important cause of poor prognosis of MuBC [20, 21]. Our research demonstrates that cancer stage is an independent prognostic factor for MuBC. The nomogram showed that the most significant contributor to prognosis was the stage. Therefore, it is particularly important to fully evaluate the cancer stages of patients with MuBC. One study showed that the majority (89%) of MuBC patients had an early diagnosis, similar to our findings [22]. We believe that this is one of the reasons why MuBC has a favorable prognosis.

Surgical treatment is the local treatment strategy for all patients with non-metastatic operable BC [23]. Different surgical procedures are selected according to tumor molecular phenotype, anatomic stage, and patient preference. One study showed that most patients with pure MuBC, including those with tumors up to 5 cm in diameter, can undergo breast-conserving surgery [24], and patients with stage T1–2 MuBC had better outcomes with breast-conserving therapy than with mastectomy [25]. For elderly patients with MuBC who cannot tolerate general anesthesia, minimally invasive surgery can even be used to remove the tumor, supplemented by endocrine therapy, and the prognosis is relatively optimistic [26]. The effect of breast-conserving surgery or mastectomy on patient outcomes was not statistically different in our study. The prognosis of patients undergoing modified radical mastectomy was worse than that of patients undergoing the other two methods, which may be related to late-stage carcinomas.

It is well known that MuBC has a good prognosis with low rates of local recurrence and distant metastasis. Case reports show that patients with MuBC who are treated surgically can achieve a good prognosis, with disease-free survival of up to 30 years, even without other adjuvant therapy [27–29]. The question of whether patients with MuBC can avoid adjuvant therapy such as chemotherapy, radiotherapy, and endocrine therapy after surgery is worthy of further investigation. Our study found that some patients who underwent surgery and chemotherapy also had risk factors that reduced their survival time, such as positive local lymph nodes and late staging. Therefore, high-risk patients should be fully evaluated to determine whether they require radiotherapy, endocrine therapy, or even targeted therapy and other postoperative adjuvant therapies. The follow-up interval for these patients should also be appropriately shortened.

Some limitations apply to our study. First, this was a retrospective study, which had deficiencies in the completeness and accuracy of information. Second, this study only analyzed patients with complete information, which resulted in a selection bias. Third, there was no subtype classification of MuBC, such as pure or mixed MuBC. Finally, hormone-related endocrine therapy and gene-related targeted therapy have received increasing attention in the treatment of malignant tumors [30–32]. But endocrine therapy, targeted therapy and other therapeutic information were left out of this study. Despite the shortcomings of this study, we validated the model internally and externally to make it more convincing. Further prospective studies are needed to validate and improve our nomogram.

## Data Availability

All data about the Surveillance, Epidemiology, and End Results (SEER) dataset is publicly available, https://seer.cancer.gov.
